# Antipsychotics and dementia in Canada: a retrospective cross-sectional study of four health sectors

**DOI:** 10.1186/s12877-017-0636-8

**Published:** 2017-10-23

**Authors:** Sebastian Rios, Christopher M. Perlman, Andrew Costa, George Heckman, John P. Hirdes, Lori Mitchell

**Affiliations:** 1 School of Public Health and Health Systems, University of Waterloo, 200 University Ave W, Waterloo, ON N2L 3G1 Canada; 2 Schlegel Research Institute for Aging, Waterloo, ON Canada; 3 Department of Clinical Epidemiology and Biostatistics and Medicine, McMaster University, Hamilton, ON Canada; 4 Winnipeg Regional Health Authority (WRHA) Home Care Program, Winnipeg, MB Canada

**Keywords:** Antipsychotics, Dementia, Home care, Long term care, interRAI

## Abstract

**Background:**

Antipsychotic medications are not recommended for the management of symptoms of dementia, particularly among persons with no behavioral or psychological symptoms. We examine patterns of antipsychotic medication use among persons with dementia across health sectors in Canada, with a focus on factors related to use among those without behavioral or psychotic symptoms.

**Methods:**

Using a retrospective cross-sectional design, this study examines antipsychotic use among adults aged 65 or older with dementia in home care (HC), complex continuing care (CCC), long-term care (LTC), and among alternate level care patients in acute hospitals (ALC). Using clinical data from January 1, 2009 to December 31, 2014, the prevalence of antipsychotic medication use was estimated by the presence of behavioral and psychotic symptoms. Logistic regression was used to identify sector specific factors associated with antipsychotic use in the absence of behavioral and psychotic symptoms.

**Results:**

The total prevalence of antipsychotic use among older adults with dementia was 26% in HC, 54% in ALC, 41% in CCC, and 48% in LTC. This prevalence ranged from 38% (HC) to 73% (ALC) for those with both behavioral and psychotic symptoms and from 15% (HC) to 31% (ALC) among those with no symptoms. The regression models identified a number of variables were related to antipsychotic use in the absence of behavior or psychotic symptoms, such as bipolar disorder (OR = 6.63 in CCC; OR = 5.52 in LTC), anxious complaints (OR = 1.54 in LTC to 2.01 in CCC), and wandering (OR = 1.83 in ALC).

**Conclusions:**

Potentially inappropriate use of antipsychotic medications is prevalent among older adults with dementia across health sectors. The variations in prevalence observed from community to facility based care suggests that system issues may exist in appropriately managing persons with dementia.

**Electronic supplementary material:**

The online version of this article (10.1186/s12877-017-0636-8) contains supplementary material, which is available to authorized users.

## Background

Behavioral and psychological symptoms of dementia are associated with functional decline and elevated risk of institutionalization [1]. A number of approaches have been recommended for managing such symptoms, including non-pharmacological interventions [2] as well as low doses of risperidone administered over limited periods of time [3]. However, there are concerns about the off label use of antipsychotic medications, particularly use among older adults not experiencing behavioral or psychological symptoms. Off label use of antipsychotic medications, depending on type and dose, is associated with increased risk of falls, heart disease, pneumonia, diabetes, cognitive decline, and, mortality among persons with dementia [4, 5]. Government, industry, and professional recommendations generally do not support the use of antipsychotics in the treatment of behavioral and psychological symptoms, except in cases of psychosis and severe aggression [6]. However, such recommendations and warnings have had minimal impact on patterns of antipsychotic use among older adults across a number of jurisdictions [7]. Quality indicators of potentially inappropriate use of antipsychotic medications are routinely reported, particularly for nursing homes [8]. Publically reporting such indicators has been found to reduce the use of antipsychotic medications among older adults with dementia [9].

Patterns of antipsychotic medication use among persons with dementia are variable across care settings and jurisdictions. This issue is most commonly understood in the context of residential care settings rather than community or acute care contexts. Across four countries, the prevalence of antipsychotic use among nursing home residents ranged from 11% in Hong Kong to 38% in Finland [10]. Using data from nursing home residents and chronic care patients across 8 European countries, initiation of antipsychotic medications was associated with cognitive impairment, dementia diagnoses, behavioral symptoms, delusions, motor agitation, conflict, emergency department visits, and use of other psychoactive medications [11]. Variations in use of antipsychotics are also related to the nursing home characteristics, including the facility rate of antipsychotic prescribing [12] and variations in psychiatric consultations [13].

Estimates of the prevalence of antipsychotic use among community dwelling older adults with dementia ranges from 10% in the UK [14] to 29% in the US [15]. Findings from England and Wales suggest that prescribing of antipsychotics among older adults in the community relates to demographics and geographic factors such a deprivation [14]. There is a need for more information about the patterns antipsychotic medication use among older adults across the healthcare continuum.

This study examines the patterns of antipsychotic medication use among older adults with dementia across four health sectors in Canada. Patterns of use are examined based on the presence of behavioral and psychotic symptoms among home care clients, patients awaiting discharge from acute hospitals, complex continuing care patients, and long-term care nursing home residents. In order to understand potential misuse of antipsychotic medications, this study examines factors related to the use of antipsychotic medications among individuals with dementia who are not experiencing behavioral or psychotic symptoms.

## Methods

This study used a retrospective cross-sectional analysis of data from long stay home care (HC) where clients are expected to receive services for at least 60 days, acute care hospitals among patients designated as Alternate Level of Care (ALC) who are awaiting placement in nursing homes, complex continuing care (CCC) hospitals which are similar to chronic care hospitals in other jurisdictions, and long term care nursing homes (LTC). The ALC designation means that the person occupying an acute care bed no longer requires the level of care that is provided but is awaiting discharge to an appropriate care setting.

### Data sources

Data were obtained from the Canadian Institute for Health Information (CIHI) Continuing Care Reporting System for CCC hospitals in Ontario and Manitoba as well as LTC homes in Alberta, British Columbia, Manitoba, New Brunswick, Newfoundland, Nova Scotia, Ontario, Saskatchewan, and the Yukon. The data are based on information from the Resident Assessment Instrument (RAI) 2.0. At the time of the analysis, the most recent data available for CCC and LTC were from January 1, 2013 to December 31, 2013. In Canada CCC hospitals provide services, similar to chronic care hospitals in other jurisdictions, for individuals who require post-acute rehabilitation, complex medical care, and specialty care services.

The Home Care Reporting System at CIHI is based on data from the RAI-Home Care (RAI-HC). The most recent data at the time of analysis included long-stay home care clients in Nova Scotia assessed from January 1, 2009 to December 31, 2009 and clients assessed from January 1, 2014 and December 31, 2014 in British Columbia, Newfoundland, Ontario, and the Yukon. Data were also available for individuals designated ALC between January 1, 2014 and December 31, 2014 in British Columbia, Newfoundland, Ontario, and the Yukon.

All data were obtained through a data sharing agreement between CIHI and the interRAI Canada research group at the University of Waterloo. All data were anonymized prior to the data being shared with the research team. Since we did not carry out primary data collection, we cannot speculate as to whether patients/clients/residents were aware their data were being used for research purposes. Consent to use these data was not required given that we conducted a secondary analysis of anonymized data that was collected and submitted to CIHI as per regular clinical practice procedures. Ethical approval of these procedures was obtained through the Office of Research Ethics at the University of Waterloo.

The RAI 2.0 and RAI-HC are designed to provide a comprehensive assessment of the person [16, 17]. Both assessments capture the same items on use of antipsychotic medication, diagnosis of dementia, and behavioural and psychotic symptoms. Data quality is routinely monitored by CIHI for each submission of data; data quality has also been confirmed through independent study, including the quality of diagnostic data [18–20]. Items from each assessment can be combined into a number of subscales, included the Aggressive Behaviour Scale (ABS) [21], Activities of Daily Living Scale (ADL) [22], Changes in Health, End-stage, Signs and Symptoms scale (CHESS) [23], Depression Rating Scale (DRS) [24], and Cognitive Performance Scale (CPS) [25]. Other data from these assessments used in this study included demographics, social relationships and supports, physical and psychiatric diagnoses, mental and physical health symptoms, functioning, and treatment variables. A full description of the items and scales from the RAI 2.0 and RAI-HC that were used in this study is available within an additional word file [see Additional file 1].

### Presence of Behavioral and psychotic symptoms

Behavioral symptoms were identified based on the presence of any one of the following behaviors in the 3 days prior to assessment: verbally abusive (e.g., cursing at others), physically abusive (e.g., shoving others), socially inappropriate (e.g., throwing feces), and resisting care (e.g., pushing caregiver away during ADL assistance). Psychotic symptoms included the presence of hallucinations or delusions in the 3 days prior to assessment. A single variable was created to identify behavioral and psychotic symptoms (BPS) as no behavior or psychotic symptoms present, only behaviors were present, only psychotic symptoms were present, and both behaviors and psychotic symptoms were present.

### Prevalence of antipsychotic medication use

The RAI-HC and RAI 2.0 assess whether the person had taken an antipsychotic medication in the 7 days prior to assessment. No specific assessment is made of the class or dose of medication. A single assessment was selected for each person based on the assessment that was closest to June 1 for the calendar year of the data. Prevalence was calculated separately for each health sector as the percentage of persons who had used an antipsychotic within the 7 days prior to assessment. Prevalence was stratified by the presence of BPS using the following categories: no BPS, psychotic symptoms, behavioral symptoms, and both BPS.

### Sample

The sample included adults aged 65 or more with a dementia diagnosis. There were 2.5% (*n* = 2539) of the LTC sample and 1.7% (*n* = 56) of the CCC sample that were excluded based on the presence of a diagnosis of schizophrenia while <1% in each sample was excluded based on the presence of Huntington’s disease, where antipsychotic use is often appropriate [26]. These exclusions could not be extended to ALC and HC data, as specific psychiatric diagnostic information were not available for these settings. This resulted in 40,650 persons in HC, 7477 persons in ALC, 4318 in CCC, and 90,846 in LTC. [See Additional file 2] for a table with the sample sizes and timeframes for data used in this study by Province/Territory.

### Data analysis

Logistic regression was used to examine factors associated with antipsychotic use for those without BPS in each health sector. Non-parametric tests, such as chi-square, were used to identify bivariate relationships between antipsychotic use and demographics, diagnoses, clinical characteristics, and interventions chosen based on clinical relevance from the literature. A *p* value of less than 0.01 was considered statistically significant and confidence intervals were also examined to assess the strength of effect sizes. The c-statistics (area under the ROC curve) of the models were used to interpret the strength of the models in discriminating between those with and without antipsychotic medication use [27]. All analyses were conducted using SAS software version 9.4.

## Results

Table 1 shows the clinical characteristics of adults aged 65 and older with dementia in each health sector. The patterns of these characteristics across sectors were consistent, on average, with what would be expected in care settings that increase in intensity. For example, the proportion of persons with moderate to severe cognitive impairment based on a CPS score of 3 or more increased from 43% in home care to 79% in LTC. Similarly, the mean CHESS scores, a measure of health instability, were highest in ALC and CCC patients. Lower levels of aggressive behavior were found in HC and ALC compared to CCC and LTC. The prevalence of BPS was 4.9% in HC, 6.4% in ALC, 10.5% in CCC, and 4.6% in LTC. Across all sectors, 57% did not experience any BPS while 39% experienced behavioral symptoms, 11% experienced psychotic symptoms, and 7% experienced both BPS.

**Table 1:** Descriptive characteristics of adults age 65 or older with dementia, by health sector

	Sector			
Characteristic	HC	ALC	CCC	LTC
Mean Age (SD^a^)	84.3 (7.0)	84.4 (7.0)	84.5 (7.1)	85.8 (7.2)
Male	53.6% (21777)	52.9% (3958)	45.8% (1978)	30.1% (27331)
Selected Diagnoses				
Hypertension	N/A	N/A	60.5% (2613)	58.4% (52782)
Anxiety disorder	N/A	N/A	9.8% (423)	8.6% (7806)
Depression	N/A	N/A	23.1% (997)	30.9% (28078)
Bipolar Disorder	N/A	N/A	1.1% (49)	1.5% (1350)
Arthritis	N/A	N/A	31.6% (1366)	39.4% (35609)
Diabetes	22.3% (9080)	25.7% (1922)	27.5% (1187)	22.7% (20651)
Other Cardiovascular	70.2% (28554)	77.7% (5812)	25.3% (1094)	14.8% (13387)
Any psychiatric condition	40.9% (16639)	44.1% (3297)	49.8% (2152)	55.1% (50067)
Cognitive Performance Scale^b^				
Moderate to Severe Impairment	43.3% (17619)	70.8% (5291)	76.2% (4318)	77.9% (70758)
Activities of Daily Living Scale^c^				
- Mean (SD)	3.1 (3.9)	7.9 (4.5)	11.0 (4.3)	9.5 (4.6)
CHESS Scale^d^ - Mean (SD)	1.5 (1.2)	2.2 (1.1)	2.0 (1.5)	0.9 (1.1)
Aggressive Behavior Scale				
(ABS)^e^ – Mean (SD)	0.5 (1.0)	0.7 (1.4)	1.5 (2.4)	1.7 (2.4)
Mean ABS Score for those where ABS >0	2.0 (1.3)	2.2 (1.6)	3.3 (2.7)	3.2 (2.4)
Behavior and Psychotic Symptoms:				
None	70.2% (28554)	61.9% (4554)	50.4% (2174)	46.4% (42185)
Behavior	18.7% (7598)	26.6% (1989)	34.5% (1491)	47.3% (42967)
Psychotic	6.2% (2508)	6.1% (459)	4.6% (198)	1.7% (1553)
Both	4.9% (1990)	6.4% (475)	10.5% (455)	4.6% (4141)

^a^Standard Deviation

^b^The Cognitive Performance Scale is categorical and not normally distributed. A score of 3 or more is indicative of moderate to very severely impaired cognition

^c^The Activities of Daily Living Scale ranges from 0 to 16 with higher scores indicating more impairment of self-sufficiency in ADL performance

^d^The Changes in Health, End Stage Disease, Signs and Symptoms (CHESS) Scale ranges from 0 to 6 with higher scores indicating greater health instability and medical complexity

^e^The Aggressive Behaviour Scale, available in the data used for the CCC and LTC, ranges from 0 to 12 with higher scores indicating a greater number and frequency of behaviors in the 7 days prior to assessment. A modified version of the scale ranging from 0 to 8 was calculated for the HC and ALC data since the items used to calculate the scale reflect behaviour in the prior 3 days

### Prevalence of antipsychotic medication use

**Fig. 1:**
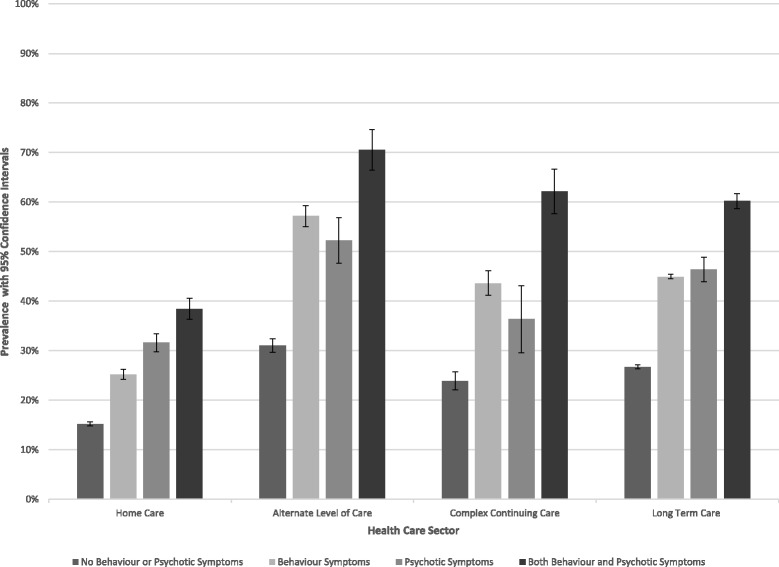
Prevalence of antipsychotic use among adults aged 65 or more with dementia within Home Care (HC), Alternate Level Care (ALC), Complex Continuing Care (CCC), & Long Term Care (LTC)

The overall prevalence of antipsychotic use among older adults with dementia was 19.2% (.95CI: 18.8%–19.6%) in HC, 41.8% (.95CI: 40.7%–42.3%) in ALC, 35.3% (.95CI: 33.9%–36.7%) in CCC, and 37.2% (.95CI: 36.9%–37.5%) in LTC. Fig. 1 provides the prevalence for each of the sectors based on symptom stratification. The figure illustrates a clear increasing trend in anti-psychotic medication use with the presence of BPS and as sectors move from community to institutional settings. In particular, prevalence of use among persons with no behaviour or psychotic symptoms rises from 12% in HC to 20% in CCC, 29% in LTC, and 32% in ALC. Considerable variability existed in the prevalence of antipsychotic use between jurisdictions. For instance, the prevalence of antipsychotic medication use ranged from 6 to 18% in HC, 27–40% in ALC, 16–24% in CCC, and 16–46% in LTC among adults with dementia and no behaviour or psychotic symptoms. Variation should be interpreted with caution due to differences in denominator sizes, with smaller denominators creating unstable prevalence. A detailed breakdown of health sector prevalence within Canadian jurisdictions can be found under Additional file 3].

### Antipsychotic medication use among adults without behaviour or psychotic symptoms

Table 2 provides the multivariate logistic regression results for each sector. Across all health sectors, factors associated with antipsychotic use among persons not experiencing BPS include wandering, anxious complaints, impaired cognitive performance, and use of antidepressants. Delirium had a strong association with antipsychotic medication use in HC and ALC, a marginal association in LTC, and an inverse association in CCC. The odds of antipsychotic medication use increased substantially based on the presence of psychiatric conditions, particularly bipolar disorder where the odds were over 5 times greater in CCC and LTC sample. The use of physical restraints in ALC, CCC, and LTC was associated with an increase in the odds of antipsychotic medication use. For CCC and LTC, unrealistic fears and repetitive physical movements were associated with increased odds of antipsychotic use. Having unstable health, based on the CHESS scale, decreased the odds of antipsychotic use in ALC, HC, and LTC; however, the odds increase slightly in CCC.

**Table 2:** Logistic regression models for antipsychotic medication use among persons who did not exhibit BPS

	Odds ratio (95% Confidence interval)			
	Home care	Alternate level of care	Complex continuing care	Long term care nursing homes
C-Statistic	c = 0.67	c = 0.69	c = 0.71	c = 0.72
Variable^a^				
Age	0.98 (0.97, 0.98)	0.98 (0.97, 0.99)		0.98 (0.98, 0.98)
Male		1.27 (1.08, 1.51)	0.89 (0.81, 0.98)	0.81 (0.79, 0.84)
Any psychiatric condition	1.54 (1.40, 1.70)			1.27 (1.23, 1.31)
Bipolar Disorder	NA	NA	5.63 (3.88, 8.17)	5.52 (5.01, 6.08)
Any neurological disorder			1.27 (1.14, 1.41)	1.56 (1.50, 1.62)
Delirium	1.34 (1.17, 1.54)	1.67 (1.40, 2.00)	0.80 (0.69, 0.93)	1.06 (1.00, 1.14)
Wandering	1.44 (1.24, 1.68)	1.83 (1.47, 2.28)	1.35 (1.18, 1.54)	1.26 (1.23, 1.29)
Anxious complaints	1.75 (1.56, 1.97)	1.77 (1.44, 2.17)	2.01(1.56, 2.59)	1.54 (1.48, 1.60)
Loss of appetite		0.67 (0.51, 0.87)		
Sad, pained, worried facial expression			0.92 (0.86, 0.99)	
Repetitive physical movements			1.21 (1.09, 1.34)	1.30 (1.26, 1.33)
Unrealistic Fears				1.30 (1.26, 1.35)
CHESS Scale	0.90 (0.86, 0.93)	0.85 (0.78, 0.92)	1.11 (1.07, 1.15)	0.94 (0.93, 0.95)
ADL Scale				0.97 (0.96, 0.97)
Conflict with others	1.18 (1.04, 1.34)	1.72 (1.35, 2.19)		
Impaired cognitive skills for daily decision making	1.21 (1.15, 1.27)	1.37 (1.25, 1.49)	1.32 (1.25, 1.40)	1.69 (1.66, 1.72)
Physical restraint	NA	1.35 (1.06, 1.72)	1.35 (1.16, 1.57)	1.25 (1.19, 1.30)
Use of Psychotropic Medications				
Anti-depressants	1.63 (1.48, 1.79)	1.45 (1.22, 1.71)	1.43 (1.30, 1.58)	1.68 (1.64, 1.73)
Hypnotics	1.32 (1.20, 1.45)			
Anti-anxiety			0.95 (0.91, 0.99)	

^a^Cells left blank indicate the variable was not significant while cells denoted as NA indicate the variable was not available for that health sector

## Discussion

This study showed that use of antipsychotics exists across the care continuum, with dramatic increases in use in institutional contexts regardless of the presence of BPS. The prevalence of antipsychotic use among home care clients in our study (26%) was similar to estimates in the U.S. but variable from international estimates. For instance, Maust et al. [15] found 28.6% of adults with dementia were prescribed antipsychotics in the U.S. while Shah et al. [14] found the prevalence of antipsychotic use was 10.1% among community dwelling older adults in England and Wales. Further variability in prevalence of antipsychotic use is noted in institutional settings. The overall prevalence in this study in CCC and LTC were similar but variability existed among LTC residents across Canadian jurisdictions [Additional file 3]. Several cross-national studies confirm the variability in prevalence in LTC. Feng et al. [10] found large variation within and between jurisdictions with estimates ranging as high as 38%, 10% lower than the prevalence identified in this study. The prevalence in this study was also higher than the prevalence estimated in LTC homes across 8 European countries [11]. Prior research in Ontario, Canada has found that the likelihood of an older adult with dementia receiving an antipsychotic in LTC is related to the overall facility-prescribing rate [12]. Therefore, the variability observed in the current study and others suggests great inconsistencies in practices within and between organizations, sectors, and jurisdictions, seemingly regardless of best practice recommendations and guidelines.

The prevalence of antipsychotic medication use among adults experiencing no BPS were surprising across health sectors. While the prevalence was lower than those with BPS, antipsychotic medications were used among adults with no BPS across health sectors with increasing prevalence in facility care settings. Use among those with no BPS was related to the presence of psychological factors and psychiatric conditions, delirium, indicators of agitation and non-aggressive behaviours, and restlessness. Given the cross-sectional design of this study it may be that use at the time of observation was the result of prior BPS or that use inherently inhibited the presentation of BPS. The association in our study between physical restraint and antipsychotic use in ALC, CCC, and LTC may support this notion. However, other variables associated with use of antipsychotics, such as disruptive, restless or anxious behaviour, cognitive impairment, and psychiatric conditions, are consistent with factors associated with the initiation of antipsychotic medication use identified elsewhere [11]. Together, these findings suggest antipsychotic medications are being used to help control the person and potentially as a preventative measure against future behaviour. While intentions for such care may not be to harm the person, the risk of medication side effects, falls, and other complications should be considered.

These results raise questions about the appropriate use of antipsychotic medications among older adults with dementia given that guidelines recommend use of specific agents over short periods for severe aggressive behaviours or psychotic symptoms [6]. Even among persons with behaviours, the mean scores on the ABS scale among those using an antipsychotic were below published thresholds for severe behaviour [28], particularly in HC and ALC. Questions also arise as to where antipsychotic prescribing begins as older adults with dementia interact with the health system. These results suggest that while there is lower use of antipsychotics in the community there is a large increase in use among individuals in transition in the health system. Individuals waiting for a LTC bed in acute hospitals had high prevalence of use both in the presence and absence of BPS. It is likely that individuals with behavioural needs who are ALC are being cared for in environments with neither the training, staffing, nor environment to fully manage or prevent behaviours. In all care settings, resources may also be limited for widespread implementation of non-pharmacological interventions to address behavioural and psychological symptoms [29]. It may be that once a person is prescribed an antipsychotic, be it in hospital or in the community, it becomes a prominent component of the person’s treatment plan leading to an accumulation of use as one transitions through the health system.

### Limitations

It was not possible to identify the type, dose, or duration of antipsychotic used. While off-label use of these medications is discouraged, the use of low doses of risperidone over short durations has been identified as an exception [3]. Given that this study uses large datasets containing clinical information collected in organizations across Canada variations in assessment approaches, and data quality, are possible. This could lead to under or overestimates of factors reported in this study. Steps to minimize such issues included the following: The assessment uses items that are mainly observational reducing issues of response bias; extensive training is provided to assessors by CIHI and through other means to ensure standard assessment processes; strict data reporting standards are established by CIHI to support data quality; data quality checks that include logic checks are performed for every submission of data to CIHI and have been confirmed through independent study [18]. It was not possible to distinguish specific types of psychiatric conditions in HC and ALC data so some persons may have had a psychotic disorder without manifesting psychotic symptoms. Further, factors at the facility and physician level were not available to distinguish variations in use of antipsychotics based on provider characteristics. Facility characteristics associated with higher use of antipsychotics have been found to include facilities that provide geriatric, pharmacist, and psychiatric care services [11]. Similarly, individuals that reside in nursing homes with high antipsychotic prescribing rates are more likely to be given antipsychotic irrespective of their clinical need [12] and have a greater mortality risk compared to residents in homes with lower rates of use [30]. Finally, comparable data were not available to examine prevalence in other health sectors such as primary care, among patients in acute hospitals not designated ALC, community psychiatry, and short-stay home care clients.

## Conclusion

Antipsychotic medication use is prevalent among older adults with dementia across several health sectors and Canadian jurisdictions. The patterns suggest a high rate of use among individuals experiencing behavior symptoms at relatively low levels. Even more discouraging is the prevalence of use among individuals not experiencing behavior and/or psychotic symptoms. The variation in prevalence observed from community to facility-based care suggests that system issues, such as provider practices and system structures, may exist in managing persons with dementia. Future research should examine provider variations in antipsychotic prescribing and use as well as geographic variations in the supply of non-pharmacological interventions for behavioural and psychological symptoms in relation to use of antipsychotic medications. Further examination is also needed to understand the patterns of antipsychotic medication use across transitions in care, particularly in relation to access to specialized care such as geriatric psychiatry consultations.

## Additional files

Additional file 1: Detailed description of variables considered in the analyses. (DOCX 16 kb)

Additional file 2: Data timeframes and numbers of adults aged 65 or older with a dementia diagnosis by setting and jurisdiction. (DOCX 13 kb)

Additional file 3: Proportion of individuals 65 years of age or older who used an antipsychotic by Province/Territory, Health Sector, and Symptom Status. (DOCX 16 kb)

## Abbreviations

.95CI: 95% confidence interval
ABS: Aggressive behaviour scale
ADL: Activities of daily living scale
ALC: Alternate level care
BPS: Behavioral and psychotic symptoms
CCC: Complex continuing care
CHESS: Changes in health, end-stage, signs and symptoms scale
CIHI: Canadian Institute for Health Information
CPS: Cognitive performance scale
DRS: Depression rating scale
HC: Home care
LTC: Long-term care
OR: Odds ratio
RAI: Resident Assessment Instrument
WRHA: Winnipeg Regional Health Authority
