# Correction to: Antipsychotics and dementia in Canada: a retrospective cross-sectional study of four health sectors

**DOI:** 10.1186/s12877-018-0819-y

**Published:** 2018-05-31

**Authors:** Sebastian Rios, Christopher M. Perlman, Andrew Costa, George Heckman, John P. Hirdes, Lori Mitchell

**Affiliations:** 1 School of Public Health and Health Systems, University of Waterloo, 200 University Ave W, Waterloo, ON N2L 3G1 Canada; 2 Schlegel Research Institute for Aging, Waterloo, ON Canada; 3 Department of Clinical Epidemiology and Biostatistics and Medicine, McMaster University, Hamilton, ON Canada; 4 Winnipeg Regional Health Authority (WRHA) Home Care Program, Winnipeg, MB Canada

## Correction

Following the publication of this article [1], the authors noticed that the results presented in the results section of the article were erroneously reported in the results section of the abstract. This correction shows both the incorrect and correct values for the results section in the abstract.

The incorrect version is:

The total prevalence of antipsychotic use among older adults with dementia was 26% in HC, 54% in ALC, 41% in CCC, and 48% in LTC. This prevalence ranged from 38% (HC) to 73% (ALC) for those with both behavioral and psychotic symptoms and from 15% (HC) to 31% (ALC) among those with no symptoms. The regression models identified a number of variables were related to antipsychotic use in the absence of behavior or psychotic symptoms, such as bipolar disorder (OR = 6.63 in CCC; OR = 5.52 in LTC), anxious complaints (OR = 1.54 in LTC to 2.01 in CCC), and wandering (OR = 1.83 in ALC).

The corrected version is:

The total prevalence of antipsychotic use among older adults with dementia was **19%** in HC, **42%** in ALC, **35%** in CCC, and **37%** in LTC. This prevalence ranged from **39%** (HC) to **70%** (ALC) for those with both behavioral and psychotic symptoms and from **12%** (HC) to **32%** (ALC) among those with no symptoms. The regression models identified a number of variables were related to antipsychotic use in the absence of behavior or psychotic symptoms, such as bipolar disorder (OR = **5.63** in CCC; OR = 5.52 in LTC), anxious complaints (OR = 1.54 in LTC to 2.01 in CCC), and wandering (OR = 1.83 in ALC).
