# Supplementary material for: Antipsychotics and dementia in Canada: a retrospective cross-sectional study of four health sectors
Source: BMC Geriatr. 2017 Oct 23;17:244. doi: 10.1186/s12877-017-0636-8 (PMC5651600; doi:10.1186/s12877-017-0636-8)
Supplement: Supplementary file 1 — Detailed description of variables considered in the analyses. (DOCX 16 kb) [file 12877_2017_636_MOESM1_ESM.docx]

**Detailed description of variables considered in the analyses**

Demographic variables were available including approximate age (only year of birth was available) and gender. Diagnostic variables included specific diagnoses as well as summary variables of diagnostic categories. An indicator of ‘any neurological disorder’ was created based on the presence of the following conditions for RAI 2.0: amyotrophic lateral sclerosis, aphasia, cerebral palsy, stroke, hemiplegia, hemiparesis, multiple sclerosis, paraplegia, Parkinson’s disease, quadriplegia, seizure disorder, transient ischemic attack, and traumatic brain injury. For the RAI-HC it included Alzheimer’s, or dementia other than Alzheimer’s, or head trauma, or hemiplegia/hemiparesis, or multiple sclerosis. Specific psychiatric diagnoses were not available for the RAI-HC. For the RAI 2.0, psychiatric diagnoses that were available included bipolar disorder and anxiety disorders. A composite variable of psychiatric conditions was created for the RAI 2.0 that included the presence of delusions or hallucinations, or a Depression Rating Scale (DRS) score of 3 or more, or a diagnosis of: anxiety disorder, or depression, or bipolar disorder in RAI 2.0. For the RAI-HC a variable called “any psychiatric” is available on the instrument and is coded based on the presence of any psychiatric disorders; this item was recoded to include the presence of delusions or hallucinations and a DRS score of 3 or more to remain consistent with the RAI 2.0.

A number of other mental health variables from the RAI-HC and RAI 2.0 were included. Anxiety symptoms were based on the presence over the prior 5 days (RAI 2.0) or 3 days (RAI-HC) of unrealistic fears, repetitive health complaints, repetitive anxious complaints such as seeking attention and reassurance regarding schedules, meals, laundry, and relationship issues. Delirium was identified on the RAI 2.0 based on the presence of any of the following behaviours in the past 7 days that appeared to be different from person’s usual functioning: easily distracted (e.g. difficulty paying attention, gets sidetracked), or periods of altered perception or awareness of surrounding, or episodes of disorganized speech, or periods of restlessness, or periods of lethargy, or mental function varies of the course of the day. For RAI-HC, delirium was defined as the presence of “sudden or new onset/change in mental function over the last 7 days including (ability to pay attention, awareness of surrounding, being coherent, unpredictable variation over course of day), or in the last 90 days client has become agitated disoriented such that his or her safety is endangered or client requires protection of others.

Individual items summarizing mood and behavioral indicators from both RAI 2.0 and RAI-HC were also assessed, including sad, pained, worried facial expressions, and wandering. These items are coded based on the frequency to which they are observed in the 3 days prior to assessment. In addition, functional indicators were also included. The presence of conflict with others for RAI 2.0 included: covert conflict with staff, or unhappy with roommate, or unhappy with residents, or openly expresses conflict/anger with family or friends. For the RAI-HC, conflict with others was coded based on the item “openly expresses conflict or anger with family/friends. Additionally, the item assessing cognitive skills for daily decision making regarding tasks for daily life in both assessments is coded as a categorical variable indicating independence, modified independence, moderately impaired, and severely impaired. Finally, a number of interventions were also included in the analysis. The use of physical restraints (trunk restraint, or limb restraint, or chair preventing rising), which was coded as used at least once in the past 7 days for RAI 2.0. For RAI-HC, physical restraint is an item which assesses if the person was physically restrained in the past 3 days (e.g limbs restrained, used bed rails, constrained to a chair when sitting). The use of other psychotropic medications was also examined, including antidepressants, hypnotics, and antianxiety medication during the last 7 days.

Using items from the RAI-HC and RAI 2.0 a number of sub-scales can be calculated to measure clinical characteristics and complexity. Described elsewhere, these included the Aggressive Behaviour Scale (ABS)[1], Activities of Daily Living Scale (ADL) [2], Changes in Health, End-stage, Signs and Symptoms scale (CHESS) [3, 4], Depression Rating Scale (DRS) [5], and Cognitive Performance Scale (CPS) [6]). Since the CPS uses categorical scoring the scale is usually dichotomized to indicate the percentage of persons scoring 3 or more, indicative of moderate to very severe impairment in cognitive performance.
