# Supplementary material for: Antipsychotics and dementia in Canada: a retrospective cross-sectional study of four health sectors
Source: BMC Geriatr. 2017 Oct 23;17:244. doi: 10.1186/s12877-017-0636-8 (PMC5651600; doi:10.1186/s12877-017-0636-8)
Supplement: Supplementary file 2 — Data timeframes and numbers of adults aged 65 or older with a dementia diagnosis by setting and jurisdiction. (DOCX 13 kb) [file 12877_2017_636_MOESM2_ESM.docx]

**Table A1: Data timeframes and numbers of adults aged 65 or older with a dementia diagnosis by setting and jurisdiction.**

| **Setting** | **Timeframe** | **Province** | **N** | **Total N** |
| --- | --- | --- | --- | --- |
| **Home Care** | Jan 1, 2009 to Dec 31, 2009 | Nova Scotia | 2661 | **40650** |
|  | Jan 1, 2014 to Dec 31, 2014 | British Columbia | 12101 |  |
|  |  | Newfoundland | 101 |  |
|  |  | Ontario | 25765 |  |
|  |  | Yukon | 22 |  |
| **Acute Hospital:**  **Alternate Level of Care** | Jan 1, 2014 to Dec 31, 2014 | British Columbia | 2121 | **7477** |
|  |  | Newfoundland | 59 |  |
|  |  | Ontario | 5289 |  |
|  |  | Yukon | 8 |  |
| **Complex Continuing Care** | Jan 1, 2013 to Dec 31, 2013 | Manitoba | 41 | **4318** |
|  |  | Ontario | 4277 |  |
| **Long Term Care Nursing Homes** | Jan 1, 2013 to Dec 31, 2013 | Alberta | 10453 | **90846** |
|  |  | British Columbia | 17480 |  |
|  |  | Manitoba | 4141 |  |
|  |  | New Brunswick | 65 |  |
|  |  | Newfoundland | 819 |  |
|  |  | Nova Scotia | 297 |  |
|  |  | Ontario | 57142 |  |
|  |  | Saskatchewan | 357 |  |
|  |  | Yukon | 92 |  |
