# Supplementary material for: Antipsychotics and dementia in Canada: a retrospective cross-sectional study of four health sectors
Source: BMC Geriatr. 2017 Oct 23;17:244. doi: 10.1186/s12877-017-0636-8 (PMC5651600; doi:10.1186/s12877-017-0636-8)
Supplement: Supplementary file 3 — Proportion of individuals 65 years of age or older who used an antipsychotic by Province/Territory, Health Sector, and Symptom Status. (DOCX 16 kb) [file 12877_2017_636_MOESM3_ESM.docx]

**Table A2: Proportion of individuals 65 years of age or older who used an antipsychotic by Province/Territory, Health Sector, and Symptom Status.**

| **Province** | **Symptoms** | **%(n) Numerator by Sector** | | | |
| --- | --- | --- | --- | --- | --- |
|  |  | **HC** | **HC-Hospital ALC** | **CCC** | **LTC** |
| Ontario | No BPS | 13.9(18121) | 27.4(3220) | 23.9(2149) | 26.6(24916) |
|  | Behavioural | 23.3(4761) | 54.2(1366) | 43.5(1477) | 44.4(29239) |
|  | Psychological | 30.2(1644) | 49.7(342) | 36.2(196) | 42.6(757) |
|  | BPS | 36.6(1239) | 69.3(361) | 62.2(177) | 58.0(2230) |
| Manitoba | No BPS | N/A^[[1]](#footnote-1)^ | N/A | 16.0(25) | 20.2(2399) |
|  | Behavioural | N/A | N/A | 50.0(14) | 37.4(1539) |
|  | Psychological | N/A | N/A | *^[[2]](#footnote-2)^ | 43.5(62) |
|  | BPS | N/A | N/A | * | 50.4(141) |
| Saskatchewan | No BPS | N/A | N/A | N/A | 37.9(190) |
|  | Behavioural | N/A | N/A | N/A | 57.3(150) |
|  | Psychological | N/A | N/A | N/A | * |
|  | BPS | N/A | N/A | N/A | * |
| Alberta | No BPS | N/A | N/A | N/A | 22.8(3976) |
|  | Behavioural | N/A | N/A | N/A | 40.8(5494) |
|  | Psychological | N/A | N/A | N/A | 45.6(186) |
|  | BPS | N/A | N/A | N/A | 60.6(797) |
| British Columbia | No BPS | 17.3(8623) | 40.1(1304) | N/A | 29.3(10000) |
|  | Behavioural | 28.7(2292) | 64.7(597) | N/A | 52.1(6135) |
|  | Psychological | 34.7(649) | 59.5(111) | N/A | 51.8(473) |
|  | BPS | 44.8(537) | 77.1(109) | N/A | 65.5(872) |
| Newfoundland | No BPS | 6.2(65) | 29.6(27) | N/A | 39.2(462) |
|  | Behavioural | 13.3(15) | 47.8(23) | N/A | 65.0(266) |
|  | Psychological | * | * | N/A | 59.6(47) |
|  | BPS | 41.7(12) | * | N/A | 68.2(44) |
| New Brunswick | No BPS | N/A | N/A | N/A | 45.7(35) |
|  | Behavioural | N/A | N/A | N/A | 42.9(21) |
|  | Psychological | N/A | N/A | N/A | * |
|  | BPS | N/A | N/A | N/A | * |
| Nova Scotia | No BPS | 17.8(1732) | N/A | N/A | 20.6(170) |
|  | Behavioural | 27.0(523) | N/A | N/A | 38.2(89) |
|  | Psychological | 33.8(204) | N/A | N/A | 58.3(12) |
|  | BPS | 32.2(202) | N/A | N/A | 84.6(26) |
| Yukon | No BPS | * | * | N/A | 13.5(37) |
|  | Behavioural | * | * | N/A | 38.2(34) |
|  | Psychological | * | * | N/A | * |
|  | BPS | * | * | N/A | 68.8(16) |

1. N/A indicates no data were available. [↑](#footnote-ref-1)
2. The symbol * indicates any cell with less than 10 subjects was omitted from the table to protect confidentiality [↑](#footnote-ref-2)
